# Inhalation toxicity of indoor air pollutants in *Drosophila melanogaster* using integrated transcriptomics and computational behavior analyses

**DOI:** 10.1038/srep46473

**Published:** 2017-06-16

**Authors:** Hyun-Jeong Eom, Yuedan Liu, Gyu-Suk Kwak, Muyoung Heo, Kyung Seuk Song, Yun Doo Chung, Tae-Soo Chon, Jinhee Choi

**Affiliations:** 1 School of Environmental Engineering, University of Seoul, 163 Siripdaero, Dongdaemun-gu, Seoul 02504, Korea; 2 The Key Laboratory of Water and Air Pollution Control of Guangdong Province, South China Institute of Environmental Sciences, the Ministry of Environment Protection of PRC, Guangzhou 510065, China; 3 Department of Biological Sciences, Pusan National University, Busandaehak-ro 63 beon-gil, Geumjeoung-gu, Busan 46241, Korea; 4 Department of Physics, Pusan National University, 2 Busandaehak-ro 63 beon-gil, Geumjeoung-gu, Busan 46241, Korea; 5 Toxicity Evaluation Center, Korea Conformity Laboratories (KCL), 8, Gaetbeol-ro 145beon-gil, Yeonsu-gu, Incheon, 21999, Korea; 6 Department of Life Science, University of Seoul, 163 Siripdaero, Dongdaemun-gu, Seoul 02504, Korea

## Abstract

We conducted an inhalation toxicity test on the alternative animal model, *Drosophila melanogaster*, to investigate potential hazards of indoor air pollution. The inhalation toxicity of toluene and formaldehyde was investigated using comprehensive transcriptomics and computational behavior analyses. The ingenuity pathway analysis (IPA) based on microarray data suggests the involvement of pathways related to immune response, stress response, and metabolism in formaldehyde and toluene exposure based on hub molecules. We conducted a toxicity test using mutants of the representative genes in these pathways to explore the toxicological consequences of alterations of these pathways. Furthermore, extensive computational behavior analysis showed that exposure to either toluene or formaldehyde reduced most of the behavioral parameters of both *wild-type* and mutants. Interestingly, behavioral alteration caused by toluene or formaldehyde exposure was most severe in the *p38b* mutant, suggesting that the defects in the p38 pathway underlie behavioral alteration. Overall, the results indicate that exposure to toluene and formaldehyde via inhalation causes severe toxicity in *Drosophila*, by inducing significant alterations in gene expression and behavior, suggesting that *Drosophila* can be used as a potential alternative model in inhalation toxicity screening.

Indoor air quality is a significant health and environmental concern because people in modern societies spend most of their time indoors, and air pollutants such as volatile organic compounds (VOCs) occur more frequently and at higher concentrations indoors than in the outdoor air. Indoor VOCs include highly toxic chemicals such as formaldehyde, acetaldehyde, benzene, toluene, and perchloroethylene. Among the VOCs, toluene and formaldehyde are frequently detected in workplaces and homes. Exposure to VOCs may result in a wide range of illnesses. Most research on indoor air pollution, however, has been based on the measurement of VOCs in residential indoor/outdoor environments and human samples. Inhalation toxicity studies using animal models are very limited and have been conducted mostly using rodent models^1^,^2^. However, it is difficult to use rodent models routinely in screening-level indoor air pollution monitoring.

The fruit fly *Drosophila melanogaster* is one of the most extensively studied alternative model organisms. The *Drosophila* model at various development stages, such as embryo, larva, and adult fly, has been used to test the toxicity of chemicals, including heavy metals, industrial VOCs, and anesthetic gases^3^,^4^. The fly model has advantages over other animal models, particularly in air pollution monitoring. In combination with behavior analysis and airborne agents, fruit flies have been used to explore the mechanisms^5^ and genetics underlying the susceptibility to ethanol intoxication, to quantify the changes in the metabolic rate during exposure to components of gasoline^6^, to document the olfactory avoidance behavior^7^, and to identify neurotransmitter pathways affected by volatile fungal toxins^8^.

The behavior depends upon the integrated processes at the subcellular, cellular, and organism level, and thus, is susceptible to disruption by a broad spectrum of chemicals and environmental stresses. Therefore, behavioral changes are considered an important indicator of chemical toxicity because it reflects the integrated physiological alteration. Behavioral changes in small animals (i.e., water flea, *Daphnia magna*) due to chemical and/or environmental stresses have been investigated in environmental toxicity tests in continuous water quality monitoring^9^,^10^.

In the present study, the inhalation toxicity of toluene and formaldehyde was investigated via transcriptomics and computational behavior analyses using the alternative model, *Drosophila*, to identify potential hazards of major indoor air pollutants. All toxicity tests were conducted in an inhalation chamber because inhalation is the main route of absorption of indoor air pollutants. Microarrays and ingenuity pathway analysis (IPA) were used to obtain insights into genome-wide responses to toluene and formaldehyde in *Drosophila*. To investigate the toxicological consequences of gene expression changes, we then analyzed the susceptibility of mutant flies that are defective in pathways overrepresented based on analysis of the transcriptome in response to toluene and formaldehyde exposure. These pathways include immune response, stress response, and metabolism. Finally, extensive computational behavior analysis was performed on *wild-type*, and *p38b* and *p53* mutant flies selected based on the transcriptome analysis and susceptibility test. Understanding the relationship between gene expression and behavior in response to VOC toxicity in *Drosophila* will help to determine the likely hazards of indoor air pollutants and to establish the potential of *Drosophila* as an alternative model for inhalation toxicity screening.

## Results and Discussion

### Inhalation toxicity of toluene and formaldehyde in Drosophila

An inhalation exposure system, which utilizes inhalation as the main route of exposure, is used to test the toxicity of chemicals. Most inhalation toxicity studies have been conducted on rodent models. In this study, we applied the system to an alternative model species, *Drosophila,* which represents an excellent model system to study the molecular mechanisms of responses to atmospheric pollutants. Although flies do not have lungs, their airway systems have enough similarities with the respiratory system of mammals to be useful model organisms for inhalation toxicity tests^11^,^12^.

**Figure 1:**
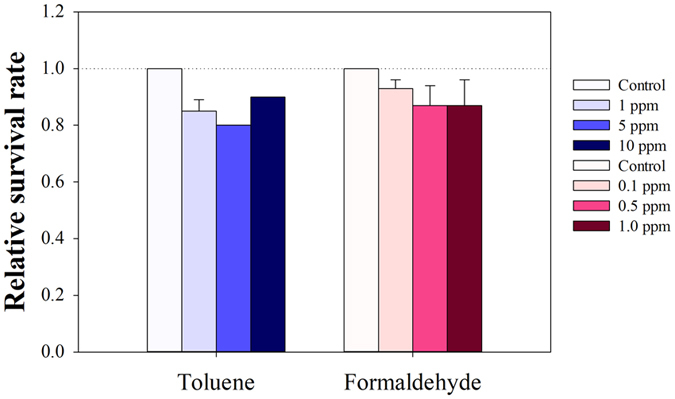
The survival test was conducted on the *wild type* exposed to toluene (**A**) and formaldehyde (**B**). The results were expressed as mean values compared with the control sample (untreated sample; control = 1, n = 3, 20 flies per replicate; mean ± standard error of the mean). The statistical analysis was conducted using two-tailed t-test, *p < 0.05.

To identify the potential hazards of toluene and formaldehyde, we first conducted a lethal toxicity test. Considering that the primary route of exposure to VOCs is inhalation, *Drosophila* was exposed to 1, 5, and 10 ppm toluene and 0.1, 0.5, and 1.0 ppm formaldehyde in an atmospheric chamber for 72 h (see Materials and Methods). The temperature and humidity in the inhalation chamber were within the range of the pertinent OECD test guidelines. The inhalation chamber system is presented in Supplementary Figure S1. VOC concentrations in each exposure chamber were maintained within the range of target concentrations throughout the experiment (Supplementary Table S1). The survival rate of *wild-type* flies decreased by 20% and 10% when exposed to toluene and formaldehyde, respectively (Fig. 1), suggesting that the exposure concentrations had limited organism-level effects.

### Transcriptomics response of Drosophila to toluene and formaldehyde

Transcriptome analysis was conducted under sublethal exposure conditions (i.e., 1,000 μg/m^3^ for toluene and 100 μg/m^3^ for formaldehyde) to understand the genome-wide effects of toluene and formaldehyde inhalation on the gene expression of *Drosophila*. Analysis of the microarray results using a 2-fold change as the cutoff value indicated that a total of 37 and 130 genes were differentially expressed upon toluene and formaldehyde exposure, respectively (Supplementary Table S2, GSE24845). Exposure to formaldehyde altered more genes than to toluene. Supplementary Figure S2 presents the results of the hierarchical clustering analysis. The expression pattern of toluene exposure clustered with that of the control sample, while formaldehyde showed a distinct expression pattern. Among the differentially expressed genes (DEGs), 86.5% (32/37) and 96.9% (126/130) were upregulated, while only 5 and 4 genes were downregulated by toluene and formaldehyde, respectively (Table S2). Interestingly, many immune-related genes, such as *Attacin (AttA, AttC*), *Diptericin-like protein (DptB*), *Metchnikowin (Mtk*), and *Turandot (TotA, TotC, TotM, TotX*) genes, were found in the DEGs of the flies that inhaled toluene and formaldehyde. The subsequent gene ontology (GO) analysis on the DEGs revealed that the immune response was one of the most significantly overrepresented GO terms for both toluene and formaldehyde exposure (Table 1A; see Supplementary Table S3 for the full set of GO categories). Considering that immune response organs express pathogen recognition proteins (e.g., peptidoglycan recognition proteins^13^) and antimicrobial proteins (e.g., Drosomycin) in response to pathogens^14^, it is interesting that these genes were identified in the DEG list from the microarray data for both chemicals (Supplementary Table S2). This result suggests that both toluene and formaldehyde induce immune response, even in the absence of pathogens. This result is also consistent with the findings of several previous studies, which showed that formaldehyde toxicity could influence the immune system^15^,^16^,^17^. In humans, for instance, occupational formaldehyde exposure was found to decrease white blood cell count^15^. Long-term exposure to formaldehyde at higher concentrations than the threshold limit (0.08 ppm) in mice enhanced allergen-specific immunoglobulin E (IgE) responses and NK (Natural Killer) cell activity in peripheral blood cells^16^. Inhalation of formaldehyde has been found to decrease secondary immune response in rats^17^. Immunotoxicity caused by toluene inhalation has also been documented. In an allergic mouse model, long-term exposure (6 weeks) to low-level toluene might dysregulate the allergic responses to ovalbumin^18^. Several reports by Fujimaki and colleagues revealed that long-term exposure to low-level toluene could disturb the neuroimmune crosstalk^19^. A genome-wide gene expression profile study on *Drosophila* determined several genes related to defense response, biological regulation, cell cycle, metabolic process, and circadian rhythms that were affected by toluene exposure^20^.

Another interesting GO term overrepresented in the DEGs by formaldehyde exposure is the neurological system process. The neurotoxicity caused by formaldehyde inhalation has been demonstrated in epidemiological and toxicological studies^21^. For instance, Tang *et al*.^22^ reported that formaldehyde induces neurotoxicity in PC12 cells by decreasing endogenous H_2_S generation, which protects nerve cells against oxidative stress. In animal studies, acute exposure of male rats to 5 ppm formaldehyde decreased the motor activity during exposure and increased the levels of dopamine, serotonin, and enzymatic metabolites in the hypothalamus^23^. In another study, the exposure to 1–10 ppm formaldehyde induced avoidance in mice^24^. Another study showed that exposure of rats to 2.6 or 4.6 ppm formaldehyde caused behavioral abnormalities observed in a maze task; the data also indicated possible neurotoxicity of formaldehyde exposure^25^. Some human studies have reported persistent central nervous system (CNS) symptoms and impairment resulting from occupational or non-occupational exposure to formaldehyde^26^,^27^. In addition, in humans, exposure to formaldehyde together with other solvents such as xylene and toluene can have depressant effects on CNS functions, causing epileptiform seizures^28^. However, neurotoxic effects of formaldehyde inhalation in *Drosophila* have not been reported yet. The results of our microarray experiment indicated that the *Drosophila* nervous system might also be affected by formaldehyde inhalation.

We conducted a Kyoto Encyclopedia of Genes and Genomes (KEGG) analysis to obtain insights into the biological processes affected by formaldehyde and toluene inhalation (Table 1B). The results revealed that toluene exposure led to an alteration in xenobiotic metabolism by cytochrome P450 (CYP450), drug metabolism, and starch and sucrose metabolism. Several members of the CYP450 superfamily are important for the metabolism of xenobiotics^29^,^30^, and their expression is also induced by toluene exposure^31^,^32^,^33^. Of them, the expression of CYP2E1, which is the primary enzyme in the hydroxylation of toluene^30^, increased in rat liver and peripheral lymphocytes after toluene exposure^31^. Similarly, a human study showed an increase in CYP2E1 in peripheral lymphocytes due to occupational toluene exposure^32^. Toluene exposure also induced other isoenzymes, including CYP1A1/2, CYP2B1/2, and CYP3A1 in rat liver^33^. Thus, our findings are consistent with those of previous studies, suggesting that toluene metabolism via CYP450 is conserved in *Drosophila*, reinforcing the importance of CYP450. KEGG pathway analysis for formaldehyde exposure indicated a significant change in the lysosome process, involved in innate immune responses and amino sugar and nucleotide sugar metabolism, which is a part of the carbohydrate metabolism for regulating protein synthesis. These findings suggest that active protein synthesis is required for the immune system to function in the defense against inhaled formaldehyde.

**Figure 2:**
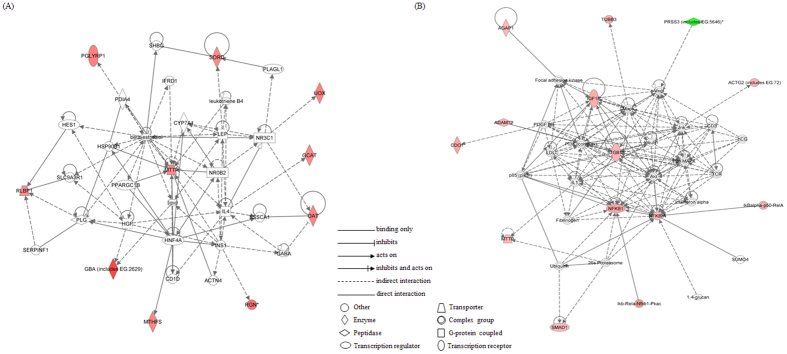
Ingenuity pathway analysis (IPA) network. The IPA was performed on DEGs to determine the toluene (**A**) and formaldehyde (**B**) exposure. A cutoff value of 1.5-fold was used for IPA.

Transcriptomics profiling generates a large amount of information, with no comprehensive mechanistic understanding provided, which makes it difficult to interpret the data. We thus performed pathway analysis using IPA to put the gene expression data in a relevant biological context. IPA revealed that nuclear factor-kappaB (NF-κb), Phosphoinositide 3-kinase (PI3K) and mitogen-activated protein kinases *p38 (p38* MAPK) are hub molecules in formaldehyde-induced toxicity, whereas MTTP and beta-estradiol are highly linked to toluene exposure (Fig. 2). In mammals, MTTP is not only essential for the assembly and secretion of apolipoprotein B (apoB)-lipoprotein, but is also involved in the immune response via its participation in the biosynthesis of CD1 proteins. The main function of CD1 proteins is to provide foreign lipids with certain T lymphocytes that affect both innate and adaptive immune responses^34^. The *Drosophila* MTTP protein is also involved in the secretion of the apoB-family lipoprotein Lipphorin (LPP), which is the major hemolymph lipid carrier^35^. Although an immune-related function of MTTP has never been reported in *Drosophila*, an unexpected but interesting finding revealed a crucial role of MTTP in tracheal development, probably through the regulation of de novo apical membrane delivery to the existing lumen, instead of its well-known function of lipoprotein secretion^36^. Thus, MTTP might participate in the maintenance or protection of membrane integrity against inhaled toluene in tracheal epithelia. Among the formaldehyde-induced genes, *p38* and *NF-κb* are the most interconnected nodes of the network (Fig. 2B). In agreement with the GO analysis, the clustering of the biological process indicates the involvement of the immune response and toll-signaling pathway in formaldehyde-induced response (Table 1). Considering that the MAPK pathways, which include *p38* and *JNK*, play pivotal roles in both stress response and innate immune response in *Drosophila*^37^, our findings suggest that formaldehyde inhalation induces crosstalk between stress response and innate immunity in *Drosophila*.

### Susceptibility of mutant flies to toluene and formaldehyde exposure

**Figure 3:**
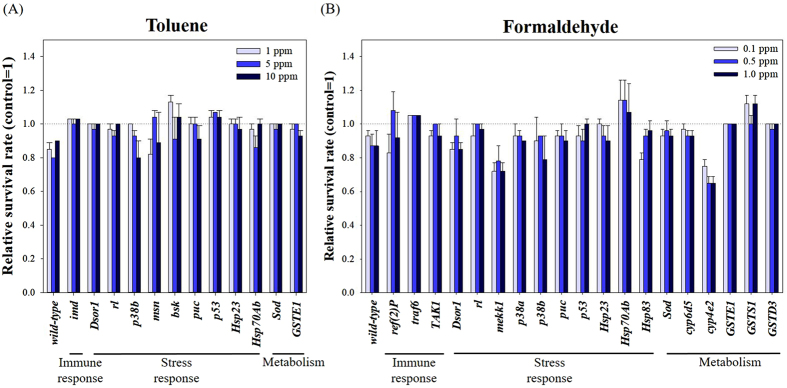
The survival test was conducted on mutants with respect to immune response, stress response, and xenobiotic metabolism exposed to toluene (**A**) and formaldehyde (**B**). The results were expressed as mean values compared with the control sample (untreated sample; control = 1, n = 3, 20 flies per replicate; mean ± standard error of the mean). The statistical analysis was conducted using two-tailed t-test, *p < 0.05.

To further investigate the toxicological consequences of altered gene expression on the immune response, stress response, and metabolism pathways, the effects of toluene and formaldehyde on living flies of representative mutant strains of each biological pathway were examined (Supplementary Table S4). Mutant flies were exposed to toluene and formaldehyde under the same conditions as the *wild-type* flies, and their survival rates were compared (Fig. 3). Counter-intuitively, the immune response-related mutants appeared to be unaffected by toluene and formaldehyde. The stress response-related mutants did not show significant alterations as compared to the *wild-type* flies, except for a few mutant strains. A concentration-dependent decrease in the survival rate of *p38b* mutants caused by toluene exposure was observed. Another alteration was determined in *mekk1* mutants; their survival rate decreased by more than 20% upon formaldehyde exposure. Mutants of xenobiotic metabolism did not show any significant alterations as compared to the *wild-type*, except for the *cyp4e2* mutant with significantly increased mortality due to formaldehyde exposure. In summary, we did not detect any significantly increased susceptibility to either toluene or formaldehyde in immune response mutants; however, some mutants of MAPK-signaling (*p38b, mekk1*) and xenobiotic metabolism (*cyp4e2*) were considerably more sensitive to toluene or formaldehyde than the *wild-type* flies. However, notably, some stocks are homozygous with respect to the mutant allele and thus, are *bona fide* loss-of-function mutants, whereas others are either heterozygous or the function of the affected gene has not yet been proven to be defective (Supplementary Table S4). Therefore, even if the sensitivity of the mutant stocks to VOCs does not significantly change, this does not necessarily mean that corresponding genes are not involved in the toxicity of VOCs. Another point to note is that we used mutant stocks without isogenization because this was a pilot study to test the *Drosophila* model as an indoor air pollutant monitoring system and the resources were quite limited, hindering the isogenization of all strains. Therefore, the contribution of genetic background to the results cannot be ruled out.

### Effect of toluene and formaldehyde on the Drosophila p38b and p53 gene expressions

The neurotoxic effects of toluene and formaldehyde have been widely reported in mammalian model organisms and epidemiological studies^19^,^26^,^28^,^38^. However, our transcriptomics analysis failed to provide direct evidence of the neurotoxic effects of formaldehyde or toluene in *Drosophila*. Nonetheless, considering that both formaldehyde and toluene can induce genotoxicity and cellular stress response, mediated by p53 and p38 MAPK, respectively, and these signaling pathways can induce cytotoxicity, the two signaling pathways might be involved in the neurotoxic effects of both formaldehyde and toluene. Indeed, several studies have demonstrated that the p53 and p38 MAPK pathways are involved in neurodegenerative diseases and neural plasticity^39^,^40^. In addition, our transcriptomics analysis and susceptibility test consistently suggested the involvement of the p53 pathway in formaldehyde-induced responses at the cellular and the organism level. Therefore, to examine the neurotoxic effects of formaldehyde and toluene inhalation in *Drosophila*, we investigated the p53 and p38 pathways in more detail using behavioral analysis.

We checked the expression levels of *p38b* and *p53* before behavior analysis. Although the two genes themselves were not detected as DEGs in our microarray experiments, they might both be induced by VOCs, because the GO and KEGG pathway analyses (Supplementary Table S3) and IPA (Fig. 2) implied an overrepresentation of pathways related to them. Therefore, we examined whether both toluene and formaldehyde could induce their expression upon higher exposure concentrations than those used in the microarray experiments (Supplementary Fig. S3). The results showed that the expression levels of both *p38b* and *p53* were significantly elevated by 0.5 ppm formaldehyde (Supplementary Fig. S3B). Toluene also induced both genes, although it was less effective than formaldehyde (Supplementary Fig. S3A). These results suggest that the *p38b* and *p53* genes can indeed be induced by both toluene and formaldehyde in *Drosophila*.

### Behavioral effect of toluene and formaldehyde: Movement parameters

To investigate whether inhalation of formaldehyde or toluene causes a neurotoxic effect in *Drosophila* and if the effect is mediated by p38 or p53 pathways, we systematically analyzed the behavioral responses of mutant flies, defective in *p38b* and *p53*, to VOCs. The movement tracks of the flies were monitored before and after the exposure to toluene and formaldehyde in the behavior analysis. Parameters were selected to characterize the shapes of instantaneous movements responding to chemical treatments. The speed (mm/s, movement distance divided by the observation time), acceleration (mm/s^2^, speed difference divided by time), and stop duration (total duration without movement) represent the general linear activity of the test organisms; less than 1/10 of the body size (0.1 mm) (see Materials and Methods). The locomotory rate (mm/s) was additionally measured to show how fast the test organisms move. The speed indicates the average movement distance during the total observation time, while the locomotory rate is the average movement distance when the organisms move, excluding the total duration of the stop time. The meander (rad/mm, angular change divided by time) was added to reflect the turning behavior of the test organisms. We also included the slipping number (*n*, the total number of slips), a typical response behavior, because flies slipped down toward the ground in the vertical arena in some treatment cases (see Materials and Methods).

**Figure 4:**
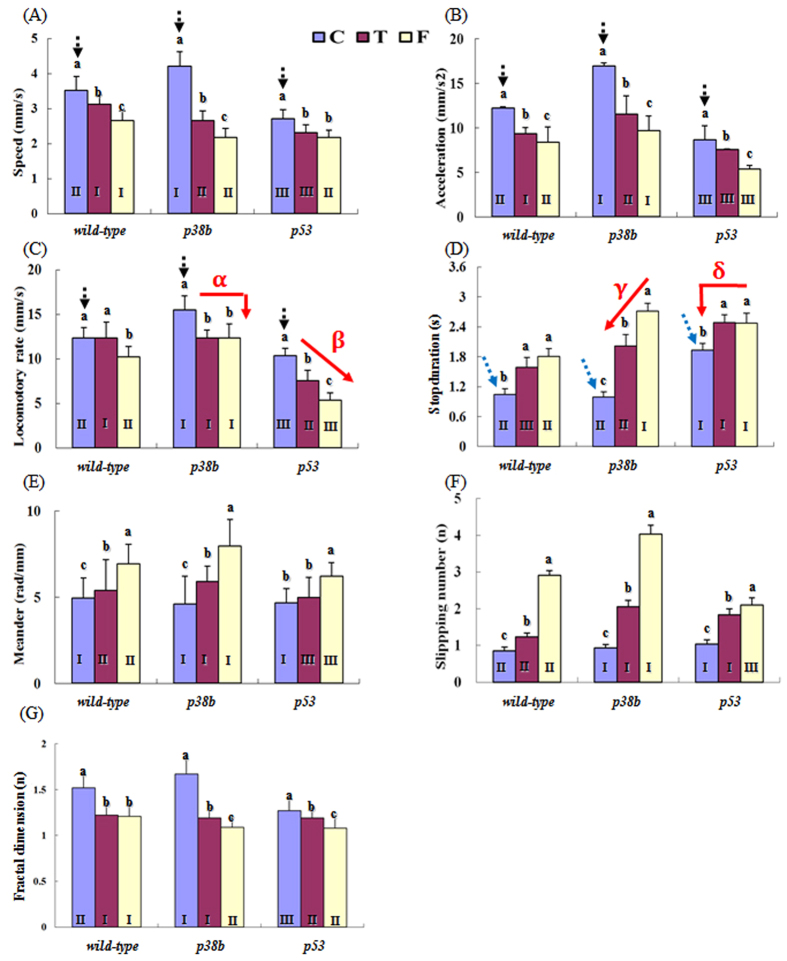
Mean and standard deviation of the movement parameters of different strains of *Drosophila* after treatment with toluene and formaldehyde (n = 15). Speed (**A**), Acceleration (**B**), Locomotory rate (**C**), Stop duration (**D**), Meander (**E**), Slipping number (**F**), and Fractal dimension of movement tracks (**G**). C: Control; T: Toluene; F: Formaldehyde. Different letters at the top of the bars indicate a significant difference between the chemicals within the same strain and different Roman numerals at the bottom of the bars shows a significant difference between the strains for the same chemical (*p* < 0.05) according to the multiple comparison tests.

Before treatments, the *p38b* mutant was most active compared to the other strains, *wild-type* and *p53* mutant flies. The speed, acceleration, and locomotory rate were generally the highest in the *p38b* mutant (*p* < 0.05, multiple comparison; compare the bars indicated by the black dotted arrows in Fig. 4A–C) before exposure to the chemicals. The mutant *p38b* showed the highest speed with 4.22 ± 0.41 mm/s, whereas the *wild-type* and *p53* mutant flies moved slowly, with speeds of 3.52 ± 0.40 mm/s and 2.72 ± 0.25 mm/s, respectively (Fig. 4A). In contrast, the stop duration was the longest in the *p53* mutant compared to that of *wild-type* and *p38* mutant flies (compare the bars indicated by blue dotted arrows in Fig. 4D). The meander and slipping number of the strains were not much different before exposure to the chemicals (Fig. 4E and F).

The parameters related to linear activity, such as speed, acceleration, and locomotory rate, decreased significantly in all strains after chemical treatment (Fig. 4A–C). In contrast, parameters more delicately representing the behavior, such as meander, stop duration, and slipping number strongly increased in all experimental groups after formaldehyde exposure (Fig. 4D–F). The speed of *wild-type* flies, for instance, decreased to 2.66 ± 0.22 mm/s after formaldehyde exposure (from 3.52 ± 0.40 mm/s), whereas it decreased to 3.12 ± 0.27 mm/s after exposure to toluene (*p* < 0.05, multiple comparison).

The *p38b* mutant was more sensitive to the chemicals than the other strains. The speed of *p38b* mutant (4.22 ± 0.41 mm/s) greatly decreased after VOC exposure: 2.66 ± 0.26 mm/s and 2.18 ± 0.18 mm/s for toluene and formaldehyde, respectively (*p* < 0.05, multiple comparisons; Fig. 4A). The two-factor analysis of the variance showed the significance of the variation in speed values between the strains (DF = 2, *p* < 0.001 with F = 965.8), between VOCs (DF = 2, *p* < 0.001 with F = 21,352), and due to interactions between the strain and VOC (DF = 4, *p* < 0.001 with F = 549.0).

The speed and locomotory rate depend on both the chemicals and the strains. While the *p38b* mutant showed the highest locomotory rate (*p* < 0.05; multiple comparison; black dotted arrows in Fig. 4C), the stop duration was significantly shorter in the *p38b* mutant than in the *p53* mutant before exposure to the chemicals (*p* < 0.05; multiple comparison; blue dotted arrows in Fig. 4D). Since *p38b* mutant had the highest values of speed and acceleration (black dotted arrows in Fig. 4A,B), it was, in general, more active than the *p53* mutant. However, the locomotory rate of the *p53* mutant was more strongly affected by the treatment (red arrow β in Fig. 4C) than that of the *p38b* mutant (red arrow α in Fig. 4C). Toluene was more effective in decreasing the locomotory rate of the *p53* mutant than formaldehyde (*p* < 0.05; multiple comparison). In contrast, the stop duration of the *p38b* mutant was more strongly affected (red arrow γ in Fig. 4D) than that of the *p53* mutant (red arrow δ in Fig. 4D). In general, the results indicate varying degrees of effects of different chemicals on distinct behavior parameters of different strains. The stop duration of *p38b* mutant was a more sensitive parameter affected by the chemicals, whereas the locomotory rate was more sensitive for the *p53* mutant. The underlying mechanisms through which the p38 and p53 pathways regulate the specific behavior parameters depend on the type of inhaled chemicals; however, to the best our knowledge, these mechanisms have not yet been clearly identified to date.

We also analyzed the slipping number, which is defined as the number of flies that slip per 30 s. The slipping number was dependent on the strains and the chemicals (Fig. 4F). The slipping number of the *wild-type* flies was 0.85 ± 0.10 (during a 5-s period) before VOC exposure but increased to 1.23 ± 0.13 and 2.91 ± 0.28 after exposure to toluene and formaldehyde, respectively. This indicated that the nervous system controlling the coordinated movement was affected by VOC exposure. The increase in the slipping number was most notable in the *p38b* mutant when treated with formaldehyde (from 0.94 ± 0.09 to 4.03 ± 0.48). Toluene also increased the slipping number to 2.05 ± 0.25 in the *p38b* mutant. However, the slipping number of the *p53* mutant was not very sensitive to VOCs compared to that of the *p38b* mutant; it increased from 1.04 ± 0.11 to 1.83 ± 0.20 (toluene) and to 2.09 ± 0.20 (formaldehyde). This suggests that the p38 signaling pathway might be involved in VOC-induced neurotoxicity that affects the coordinated behavior.

The general complexity of the movement data was further measured using the fractal dimension based on the box-counting method^41^ (Fig. 4G; see Materials and Methods). Fractal dimensions are widely used to measure complexities that appear in natural patterns with self-similarity properties (e.g., snowflakes, coastal lines). While the dimensions of area and volume are integers, 2 and 3, respectively, the fractal dimensions are fractional numbers, which originate from the uneven usage (or filling) of space. We observed the movement tracks of the test organisms in two dimensions. If the fractal dimension of the trace is close to two, it means that the fly evenly searches and passes by all of the space. However, a fractal dimension <2 indicates that the movement pattern of the fly covers certain positions. A fractal dimension close to one implies that the movements of the fly are likely linear. The fractal dimension was higher in *wild-type* (1.52) and *p38b* mutant (1.67) compared with *p53* mutant (1.27) before treatment, while it decreased substantially in *wild-type* (1.21) and *p38b* mutant (1.09) and slightly in *p53* mutant (1.08) after treatment with formaldehyde. The parameter was more strongly affected by formaldehyde (1.08–1.21) than by toluene (1.19–1.22; Fig. 4G). In general, a decrease in the complexity of the movement data was observed after treatment. The general response patterns of the fractal dimension are similar to those of the speed and acceleration (Fig. 4A,B).

The Pearson correlation coefficients of the parameters are provided in Supplementary Table S5. Significant positive correlations were observed between speed, acceleration, and locomotory rate, while speed was strongly negatively-correlated with stop duration. The fractal dimension showed a strong positive correlation with speed (*r* = 0.96) and a negative correlation with stop duration (*r* = −0.90). The meander was highly correlated with the slipping number (*r* = 0.96) because inconsistent movement due to the sharp drop caused sudden increase in slipping distance, resulting in the high level of angle change per unit time in calculating meander (rad/mm). The locomotory rate showed a stronger positive correlation with acceleration (*r* = 0.90) than speed (*r* = 0.75), while stop duration had stronger negative correlation with speed (*r* = −0.95) than with acceleration (*r* = −0.74).

Most parameters used in this study mainly represent the instantaneous movements, except for the fractal dimension. We focused on the instantaneous behavior of the test organisms over relatively short distances in a confined cage (Fig. 4). Either the field or an *in situ* small-sized arena in the laboratory would be more feasible for monitoring the diverse behavior patterns in practice. We also demonstrated that subtle behavior differences in response to different treatments could be reliably identified, providing a useful system to infer the physiological responses through behavior analysis.

In general, the results indicated that toluene and formaldehyde might lead to reduced activity of flies, at the same time causing higher turning tendencies (i.e., meander) after treatment. A similar finding has been recently reported, where flies that inhaled toluene showed sedation in a dose-dependent manner^42^. However, the potency of toluene is somewhat different between the two studies; no notable change in activity was observed at 100 ppm in the study of Tatum-Gibbs and colleagues^42^, whereas we detected changes in all behavior parameters at much lower concentrations of toluene. This discrepancy might be caused by the differences in the measuring methods. In the study of Tatum-Gibbs and colleagues, the temporal resolution of the move count was 10 min, much longer than the time window of 0.25 s used in this study. In the present study, the *p38b* mutant showed a much higher susceptibility to formaldehyde, which implied that the p38 signaling pathway might be involved in the cellular mechanisms underlying the formaldehyde-induced neurotoxicity.

### Behavioral effect of toluene and formaldehyde: Duration of stay in defined areas

In addition to movement parameters, we also analyzed another behavior parameter that might be specifically related to the effect of VOCs; the duration of stay in defined areas in 30 s. The total duration of stay in a specific area in the arena also varied with the strain type and VOCs (Supplementary Fig. S4). The *p53* mutant spent less time in the top area of the arena than both *wild-type* and *p38b* mutant flies before exposure, implying a less active negative-geotactic behavior (Supplementary Fig. S4A). Exposure to both VOCs increased the duration of stay in the top area for all three strains. The *p38b* mutant showed the highest value of the slipping number and the duration of stay in the top area after exposure to VOCs indicating that the *p38b* mutant slipped frequently but showed very active negative geotaxis and climbed up the vertical arena in response to VOC exposure. The duration of stay in the bottom area showed a reverse pattern; all three strains of flies spent less time in the bottom area after VOC treatment than before the treatment (Supplementary Fig. S4B). The duration of stay in the food area was relatively shorter than that in the top or the bottom area (Supplementary Fig. S4C). When treated with VOCs, however, both *p38b* and *p53* mutants spent less time in the food area than did *wild-type*, although the duration decreased for all three strains.

### Behavior effect of toluene and formaldehyde: Self-organizing map (SOM) analysis

**Figure 5:**
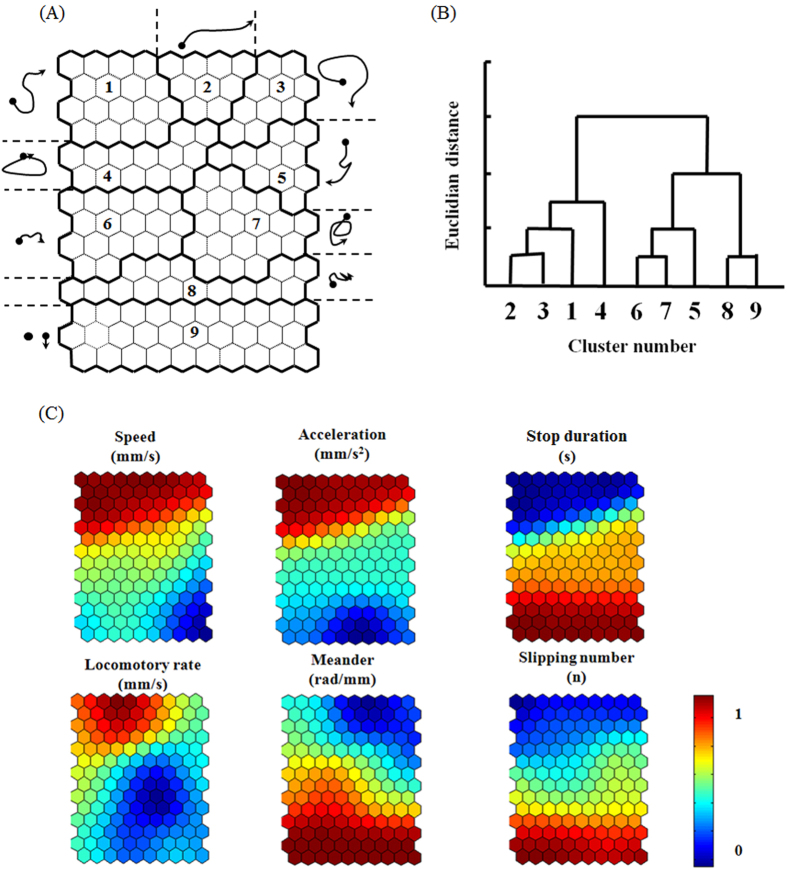
Movement patterns of *Drosophila* on the self-organizing map (SOM) of different strains after treatment with toluene and formaldehyde (n = 15). Nine clusters classified by the SOM along with representative movement patterns (**A**); dendrogram according to Ward’s linkage method (**B**); components of the weight vectors matching the clusters based on the trained SOM (**C**). The values of the vertical bars in the bottom right corner indicate the ranges of parameters normalized to 0–1.

Movement tracks were further patterned by self-organizing map (SOM) analysis based on the six input parameters representing the instantaneous movement behavior after exposure to VOCs (see Materials and Methods; Fig. 5). The fractal dimension was not included as input data because that parameter reflects the general complexity of the movement data during a longer observation period (30 s). The movement segments were grouped into nine clusters (Fig. 5A) based on the dendrogram of the Ward’s linkage method (Fig. 5B)^43^. In general, a vertical gradient was observed, indicating higher activity at the upper area and lower activity in the bottom area, as shown in the component plane visualization of the SOM (Fig. 5C). The upper clusters were subdivided into three clusters (clusters 1, 2, and 3) to show linear and round movements with high speed. Segments representing the stop or minimum movement were broadly observed in cluster 9 at the bottom of the SOM (Fig. 5A). Movements with zigzag patterns were observed in the middle area of the SOM including clusters 5, 6, and 7. The movement patterns shown in Fig. 5A were typical examples of the parameter profiles displayed in Fig. 5C.

**Figure 6:**
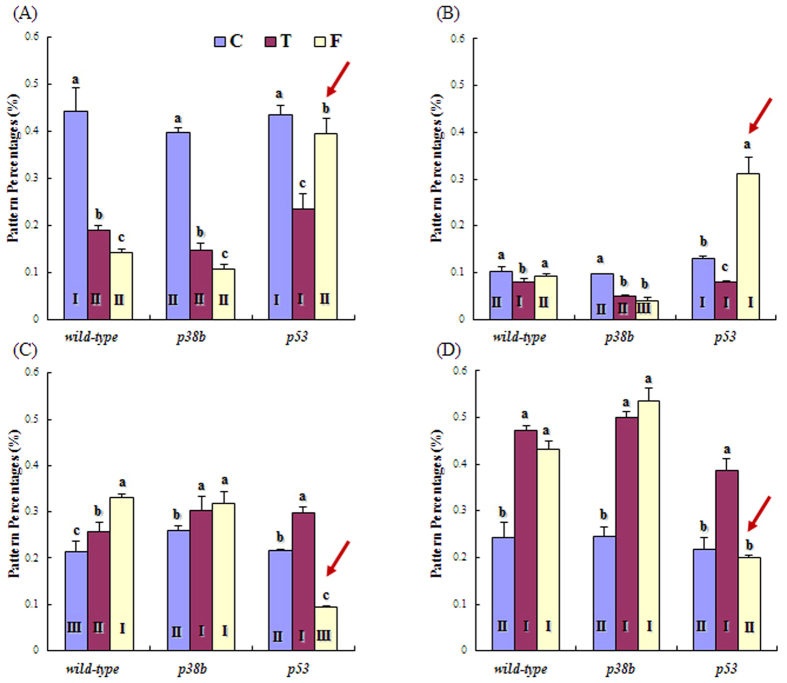
Mean and standard deviation of the movement pattern percentages when strains of *Drosophila melanogaster* were treated with toluene and formaldehyde (n = 15). P1 + P2 + P3 (**A**), P4 (**B**), P5 + P6 + P7 (**C**), and P8 + P9 (**D**). C: Control; T: Toluene; F: Formaldehyde. Different letters at the top of the bars indicate a significant difference between the chemicals within the same strain and different Roman numerals at the bottom of the bars show a significant difference between the strains for the same chemical (*p* < 0.05) according to the multiple comparison tests.

We then analyzed the characteristics of each cluster shown in the dendrogram for all strains and chemicals (Fig. 6). The nine clusters of the SOM were grouped into four general movement patterns: 1) “P1 + P2 + P3” for active movement either “from stop to movement” or “from movement to stop”; 2) “P4” for circling; 3) “P5 + P6 + P7” for zigzag; and 4) “P8 + P9” for the least movement or stop. The percentages of the grouped patterns are shown in Fig. 6 (see Supplementary Fig. S5 for the percentages of all nine movement patterns for all combinations of strains and treatments).

The active movements represented by “P1 + P2 + P3” were dominant before treatment, accounting for more than 40% of the observation time (6 h) for all strains (Fig. 6A). Circling was observed the least and accounted for slightly more than 10% of the observation time (6 h; Fig. 6B). The zigzag pattern and the least movement/stop were similar and accounted for 21–26% of the observation time (Fig. 6C,D). The percentages of the movement patterns did not vary significantly between the strains before treatment. Significant differences, however, were observed after treatment. The group “P8 + P9” indicated that the stop/least movement increased after treatment with both formaldehyde and toluene for all strains (Fig. 6D) with the exception of formaldehyde-treated *p53* mutants for which the increase was not evident (arrow in Fig. 6D). The active movements represented by “P1 + P2 + P3” decreased substantially after treatment with the same exception: the active movement pattern remained the same after the treatment of *p53* mutants with formaldehyde (arrow in Fig. 6A). Similarly, the circling pattern “P4” was uniquely present at a high level in the *p53* mutant after treatment with formaldehyde, whereas the pattern was either at the same level or slightly decreased for all strains treated with toluene and for formaldehyde-treated *p38b* mutant (arrow in Fig. 6B). The zigzag movement increased after treatment, except for the formaldehyde-treated *p53* mutant (arrow in Fig. 6D). In summary, the formaldehyde treatment of *p53* mutants differently affected their response mechanisms, whereas other cases generally showed similar behavior patterns. Formaldehyde and toluene had opposite effects on the behavior. Generally, the percentages of “P1 + P2 + P3” and “P4” were higher, while those of “P5 + P6 + P7” and “P8 + P9” were lower in formaldehyde-treated groups and reverse patterns were observed in toluene-treated groups (Fig. 6).

Our SOM analysis helped to discriminate subtle behavior differences between the strains and treated chemicals and categorize them accordingly, although the precise discrimination and categorization were not successful because of the complexity of the movement behavior. Since the behavior is an activity of the nervous system, having changed several behavioral parameters, toluene and formaldehyde affected the function or the structure (or both) of the nervous system. However, molecular mechanisms underlying the disruption of neural functions in response to VOC exposure are still unclear.

### Inhalation toxicity screening in Drosophila

We evaluated *Drosophila* as an alternative model for inhalation toxicity screening. The microarray analysis showed that inhalation of both toluene (1,000 μg/m^3^) and formaldehyde (100 μg/m^3^) for 24 h was sufficient to induce genome-wide gene expression changes in *Drosophila*. The GO and pathway analyses for DEGs identified GO terms or pathways similar to those previously reported in a murine model including GO terms of immune response, xenobiotic metabolism, neurological system process, and pathways related to the cellular stress response such as NF-κb, PI3K, and p38 MAPK pathways^15^,^16^,^17^,^18^,^19^,^20^. This indicated that the inhalation of toluene and formaldehyde induced similar cellular responses in *Drosophila* compared to those in mammals. Our behavioral assay showed that both toluene and formaldehyde could also induce substantial behavioral changes in *Drosophila*, suggesting that the behavioral changes can be used as indicators of air quality. The finding that the *p38b* mutant was more susceptible to both VOCs implies that the p38 MAPK pathway may be involved in this process and this mutant strain is a much better monitor than the *wild-type*. This result also suggests that more sensitive mutant strains can be identified by testing the strains that alter this pathway.

Because the primary goal of this study was to evaluate *Drosophila* as an alternative biomonitoring system for VOC pollution, we did not focus on the molecular and cellular mechanisms underlying the behavioral changes in response to VOC exposure. Therefore, further studies to understand the mechanisms will provide valuable information about the biology of VOC-induced neurotoxicity and efficient ways to apply the *Drosophila* model as a versatile biomonitoring system to inhalation toxicity screening.

## Materials and Methods

### Flies and maintenance

The flies were reared on standard fly food containing agar, sugar, dry yeast, cornmeal, nipagen M, and propionic acid, unless otherwise noted. All flies used in this study were obtained from the collection of the Bloomington *Drosophila* Stock Center. Detailed information on the flies used in this study is given in Supplementary Table S4.

#### Exposure conditions

**Figure 7:**
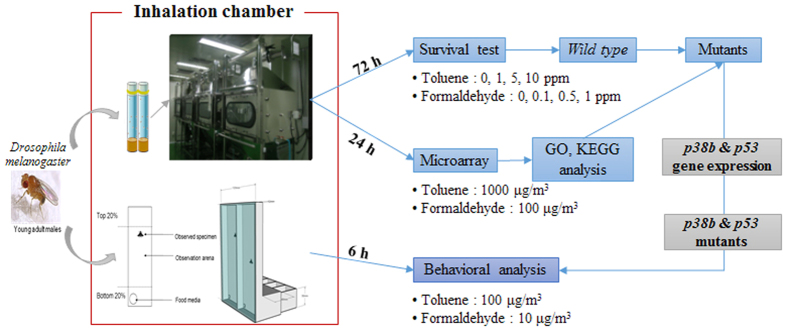
The experimental scheme of exposure to toluene and formaldehyde in *Drosophila* using an inhalation chamber.

The exposure concentrations of toluene and formaldehyde for *Drosophila* were determined based on the permissible exposure level (PEL) by inhalation. International safety and occupational health agencies have set the PELs of toluene and formaldehyde by inhalation. Most levels are based on the epidemiological and toxicological test outcomes obtained from both human and animal models for a certain exposure time or are based on health hazard assessments in the relevant toxicological literature. The occupational threshold limit values (TLV) are categorized as time-weighted average (TWA), short-term exposure limit (STEL), and ceiling (C) values, with the latter defining the exposure limit. The workplace toluene standards of 200 ppm (754 mg/m^3^) as an 8-hour PEL and 50 ppm (189 mg/m^3^) as a time-weighted average (TWA) have been recommended by the Occupational Safety and Health Administration (OSHA)^44^ and the American Conference of Governmental Industrial Hygienists (ACGIH), respectively, to protect against the effects on the central nervous system^45^. The STEL and PEL-TWA of formaldehyde have been set to 2 ppm in 15 min and 0.75 ppm by OSHA. The National Institute for Occupational Safety and Health (NIOSH) has set a more stringent STEL of 0.1 ppm and a recommended exposure limit for occupational exposure of 0.016 ppm^46^. The most common STEL for formaldehyde is 100 μg/m^3^ as a 30-min average set by the World Health Organization (WHO), considering the portal-of-entry effects and sensory irritation of eyes and upper airways^47^. Considering these PELs set by various international safety and occupational health agencies and the concentration levels in the indoor environment, the flies were exposed to 1,000 μg/m^3^ of toluene and 100 μg/m^3^ of formaldehyde for 24 h transcriptomics and 100 μg/m^3^ of toluene and 10 μg/m^3^ of formaldehyde for 6 h behavior analysis. All experiments (i.e., survival test, transcriptomics, gene expression, and behavior analysis) were conducted in an inhalation chamber and the general experimental scheme is presented in Fig. 7.

### Microarray

For microarray analysis, newly emerged adult male flies were exposed to 1,000 μg/m^3^ of toluene (Sigma-Aldrich #179418) and 100 μg/m^3^ of formaldehyde (Sigma-Aldrich #F8775) for 24 h. The total RNA prepared from the exposed and control flies is based on the standard protocol of the RNeasy Mini Kit (Qiagen, Hilden, Germany). Five-microgram aliquots of each total RNA product were used for reverse and *in vitro* transcription followed by the application to a GeneChip^®^ *Drosophila* Genome 2.0 Array (Affymetrix, Santa Clara, CA, USA), which contained 18,880 probe sets and 18,500 unique *Drosophila* transcripts. The MAS5 algorithm was used for the expression summary and the signal calculation of Affymetrix *D. melanogaster* genome array data. Global scaling normalization was performed; the normalized data was base 2 log-transformed. The fold change and Welch t-test were applied to select differentially expressed genes; the fold change threshold was 2-fold. Furthermore, the probe sets with A calls in the compared groups were removed to filter false positives (each probe set of the Affymetrix GeneChip data has a detection call: P, present call is considered good quality; M, marginal call has intermediate quality; A, absent call has relatively low reliability). A Gene Set Enrichment Analysis was performed to investigate the functional relationships between the 2-fold DEGs using the high-throughput Gene Ontology Miner. The 2-fold DEGs of relevant pathways were mapped using the GenPlex™ v2.4 software (ISTECH Inc., Korea). The pathway resources were provided by the Kyoto Encyclopedia of Genes and Genomes database.

### Ingenuity Pathway Analysis

Raw data from the Affimetrix chip CEL files were normalized using the Robust Multichip Average (RMA) implemented in R (www.R-project.org). The RMA consists of three steps: the background adjustment, quantile normalization, and summarization. Once the data was processed in R, the log 2 value of each gene was converted into the fold change value and a cutoff value of 1.5-fold was chosen for IPA. For each gene within the cutoff value, the identification (ID) number (probe #) and corresponding fold change were tabulated in Microsoft Excel and imported into IPA (Ingenuity Systems, www.ingenuity.com) to construct networks of interacting genes. IPA contains a database that uses the most current knowledge available on genes, proteins, chemicals, normal cellular and disease processes, and signaling and metabolic pathways needed for pathway construction.

### Generation, monitoring, and analysis of toluene and formaldehyde in the inhalation chamber

To ensure the accuracy of toluene and formaldehyde concentrations in inhalation chambers, the chambers were evaluated two weeks prior to the beginning of the study. The concentration in the chambers was adjusted using a flow meter to maintain the target concentration. The toluene concentrations were determined in the chambers as described in the National Institute for Occupational Safety and Health Manual of Analytical Methods (NMAM; No. 1500; “Hydrocarbons, AROMATIC”) and formaldehyde concentrations were determined based on the NIOSH Manual of Analytical Methods (NMAM; No. 2016; “FORMALDEHYDE”). The toluene sampling was performed using a model 497701 Escot Elf pump personal air sampler (MSA, Pittsburgh, PA, USA) connected to a Gemini twin port sampler (MSA) and a charcoal tube (50/100 mg). The sampler flow rate was 0.2 L/min for 1 h. The toluene in the charcoal tube was measured by gas chromatography (GC) using a model 6890 N apparatus (Agilent Technologies, Santa Clara, CA, USA). Formaldehyde sampling was conducted using a model 497701 Escot Elf pump personal air sampler (MSA, Pittsburgh, PA, USA) connected to a Gemini twin port sampler (MSA) and a 2,4-DNPH cartridge. The sampler flow rate was 0.5 L/min for 30 min. The formaldehyde in the 2,4-DNPH cartridge was measured using high-pressure liquid chromatography (HPLC, SURVEYOR, USA). The mortality tests were conducted by counting the dead flies after exposing one-day-old male flies to toluene (1, 5, and 10 ppm) or formaldehyde (0.1, 0.5, and 1 ppm) for 72 h.

### Quantitative real-time-polymerase chain reaction

For quantitative real-time-polymerase chain reaction (qRT-PCR) analysis, *wild-type* flies were exposed to 1, 5, and 10 ppm of toluene and 0.1, 0.5, and 1 ppm of formaldehyde for 72 h in an inhalation chamber. The gene expression was analyzed using IQTM SYBR Green SuperMix (Bio-Rad). The quantitative RT-PCR was carried out on selected genes using a Chromo4 Real-Time PCR detection system (Bio-Rad). The primers were designed by the online Primer-BLAST software of the NCBI and are described in Supplementary Table S6. The qRT-PCR conditions were optimized and efficiency and sensitivity tests were performed for each gene prior to the main experiment. Three biological replicates were measured in each qRT-PCR analysis.

### Behavior observation system

The movement tracks of the test organisms were recorded in two dimensions using an observation system consisting of an observation arena, a CCD camera (Hitachi KP-D 20 BU^®^), a timer, an A/D interface (Matrox Morphis^®^), and software for image recognition (0.25 s/frame; Supplementary Fig. S6). Three strains, one *wild-type* strain and two mutants (*p38b* and *p53*), were reared under standard culture conditions (temperature of 25 °C ± 1 °C and humidity of 50% ± 5%; mean ± standard deviation; SD).

A robust background subtraction algorithm based on frame differencing with a filter was used for image recognition in this study^48^. The algorithm has previously been used to observe and record whole-body movements of relatively small indicator species such as drosophila^49^, daphnia^50^, and zebrafish^51^.

Based on the previous studies of the geotaxis behavior^52^, the vertical arena (150 mm height × 50 mm width × 10 mm depth) was selected for the observation of *D. melanogaster*. The average speed of the flies in preliminary studies was 1.52 ± 0.40 mm/s without treatment. The size of the arena was equivalent to 30 and 100 movement sequences in the horizontal and vertical directions, respectively. This number of movement sequences was sufficient to present the diverse movement behavior such as the patterns expressed on the SOM (Fig. 5). The test organisms were randomly chosen from stock populations and were placed individually in an observation arena in a vertical position. Two volatile organic compounds, toluene and formaldehyde, were diluted and sprayed on the cotton placed within the observation cage at concentrations 100 and 10 μg/m^3^, respectively. Fifteen organisms of each group (three strains × three treatments) were observed separately for 6 h without treatment and for additional 6 h after treatment with different chemicals.

### Measurement of the movement parameters

The time frame to determine the position of the individuals was set to 0.25 s because we aimed to monitor general movement changes of the individuals (i.e., 2-D location) in response to the chemical treatment for a relatively long period of time (i.e., 6 h). The 0.25-s segments were sufficiently short to present the general displacement of the organisms^53^. The extremely fast behavior due to intoxication, such as compulsion and trembling, may be expressed in less than 0.25 s; however, we did not intend to record this type of behavior in this study. The total observation time (6 h before and after treatment) was sufficiently long to record the response behavior, considering the response time of the chemical treatments^51^.

To characterize the movement parameters, the general and specific activities were measured in 5-s segments comprising 20 consecutive 0.25-s sub-segments: speed (mm/s, average speed of the sub-segments), acceleration (mm/s^2^, difference between two consequent speeds), locomotory rate (mm/s, movement distance per unit time excluding stop time), meander (rad/mm, angular change per movement distance between sub-segments), stop duration (total duration without movement; less than 1/10 of body size, 0.1 mm), and slipping number (*n*, total number of slips onto the vertical arena). In addition, the total duration of stay (s) in different locations of the arena (top, bottom, and food area) was obtained during the observation period.

The general feature of the images of the movement tracks was extracted using the fractal dimension based on the box-counting method^41^.


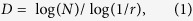


where *N* is the number of boxes with points (positions of flies in this study) and *r* is the box size. While the topological dimension of a line is 1.0 and that of a surface is 2.0, the fractal dimension may be any number between 1.0 and 2.0 because the observations were performed in two dimensions in this study. The duration of the movement tracks was set to 30 min, which was sufficiently long to differentiate the chemical effects from the behavior of other animals such as chironomids^54^.

### Self-organizing map (SOM) analysis

The self-organizing map (SOM), consisting of two layers of input and output, represents the non-linear projection of data onto a two-dimensional space and provides a patterned map of input data trained with unsupervised learning^55^. Data-driven (or heuristic) methods, including SOM, are superior to conventional statistical methods in information extraction (e.g., clustering, grouping, and associative relationships) of nonlinear data and have been extensively used in ecological sciences^56^,^57^. The size of the SOM was determined heuristically, in such a way that the variation of the movement patterns on the map are comprehensible to the reader. The optimal size was adjusted based on the degree of discrimination between the grouped nodes after training. The starting point of the node size determination is approximately 5√*n*, where *n* is the number of samples^58^. Approximately 2/3 of the total nodes may be occupied by input data, while 1/3 remains empty, which serves as a delimiter between the occupied nodes. The vertical size of the SOM was allowed to be slightly longer than the horizontal size. This way, the highest variance in the input data will be projected along the vertical axis, while the next highest variance is presented on the horizontal axis. The adjusted size of 14 × 10 nodes was used in this study.

The Euclidian distance, *d*_*j*_ (*t*), at the *j*-th node on the SOM between the weight at the iteration time *t* and the input vector was calculated using learning processes:


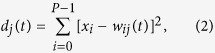


where *x*_*i*_ is the value of the *i*-th parameter, *w*_*ij*_*(t)* is the weight between the *i*-th parameter and the *j*-th node on the SOM, and *P* is the number of parameters. In this study, six parameters were used as input data for the SOM (see Measurement of Movement Parameters section).

The best matching neuron with the minimum distance was chosen as the winner. The weight vectors for the best matching neuron and its neighbor neurons were updated as:


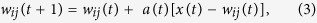


where *t* is the iteration time and *α (t*) is the learning rate. The learning process of the SOM was conducted using the SOM Toolbox developed by the Laboratory of Information and Computer Science of the Helsinki University of Technology (http://www.cis.hut.fi/projects/somtoolbox/) in Matlab environments (Mathworks, R2011)^58^. For initialization and training processes, we followed the suggestions by the SOM Toolbox allowing the optimization of the algorithm. A detailed description of the application of the SOM to ecological data is given by Park *et al*.^59^.

The weights of the best matching unit and the computation nodes close to it were adjusted towards the input vector by interactive calculations because the input data were fed to the SOM for training [Equation (3)]. A drawback, however, is that no measure could be given with respect to determining the distance between the grouped nodes after training, whereas the degree of the data variation (e.g., eigenvalue) is provided in statistical methods. To reveal the degree of association between the grouped SOM units, the Ward’s linkage method was used to cluster the movement data based on the dendrogram using the Euclidean distance matrix^48^,^49^. The linkage distances were rescaled to 0–100%. After clustering, the distance between the clusters depending on a different degree of the closeness was provided, as shown in the dendrogram (Fig. 5B).

### Data plotting and statistical analysis

Sigma Plot 12.0 was used for data plotting and statistical analysis. The statistical significances of the combined survival data were analyzed using the Survival LogRank test.

## Additional Information

**How to cite this article:** Eom, H.-J. *et al*. Inhalation toxicity of indoor air pollutants in *Drosophila melanogaster* using integrated transcriptomics and computational behavior analyses. *Sci. Rep.* **7**, 46473; doi: 10.1038/srep46473 (2017).

**Publisher's note:** Springer Nature remains neutral with regard to jurisdictional claims in published maps and institutional affiliations.

## Supplementary Material

Supplementary Information

Supplementary Table S2

## Figures and Tables

**Table 1:** The representative biological process of DEGs in *Drosophila* was generated by Gene ontology (A) and Kyoto Encyclopedia of Genes and Genomes (KEGG) pathway (B) analysis under toluene and formaldehyde exposure.

Treatment	Term	Count	%	*P* value
**(A) Gene Ontology**				
Toluene	Innate immune response	6	8.6	1.79E-05
	Proteolysis	9	12.9	0.00371
	Response to oxidative stress	3	4.3	0.02065
	Antibacterial humoral response	5	50.0	5.30E-10
Formaldehyde	Innate immune response	22	11.2	1.53E-21
	Immune response	26	13.2	1.20E-20
	Antibacterial humoral response	26	13.2	1.20E-20
	Toll-signaling pathway	7	3.6	1.51E-06
	Proteolysis	23	11.7	7.27E-05
	Response to oxidative stress	4	2.0	0.04026
	Neurological system process	4	18.2	0.01989
**(B) KEGG analysis**				
Toluene	Metabolism of xenobiotics by cytochrome P450	4	5.71	0.00398
	Drug metabolism	4	5.71	0.00435
	Starch and sucrose metabolism	3	4.29	0.03896
Formaldehyde	Lysosome	4	2.030	0.05112
	Amino sugar and nucleotide sugar metabolism	3	1.523	0.08938
