# Supplementary material for: Inhalation toxicity of indoor air pollutants in Drosophila melanogaster using integrated transcriptomics and computational behavior analyses
Source: Sci Rep. 2017 Jun 16;7:46473. doi: 10.1038/srep46473 (PMC5472918; doi:10.1038/srep46473)
Supplement: Supplementary Information [file srep46473-s1.doc]

**Supplementary Information**

**Inhalation toxicity of indoor air pollutants in *Drosophila melanogaster* using integrated transcriptomics and computational behavior analyses**

**Hyun-Jeong Eom^1,a#^, Yuedan Liu^2,#^, Gyu-Suk Kwak^2,b^, Muyoung Heo^3^, Kyung Seuk Song^4^, Yun Doo Chung^5*^, Tae-Soo Chon^2,b*^, Jinhee Choi^1*^**

^1^School of Environmental Engineering, Graduate School of Energy and Environmental system Engineering, University of Seoul, 163 Siripdaero, Dongdaemun-gu, Seoul 02504, Korea

^2^ The Key Laboratory of Water and Air Pollution Control of Guangdong Province, South China Institute of Environmental Sciences, The Ministry of Environment Protection of PRC, Guangzhou 510065, China.

^3^Department of Physics, Pusan National University, 2 Busandaehak-ro 63 beon-gil, Geumjeoung-gu, Busan 46241, Korea

^4^Toxicity Evaluation Center, Korea Conformity Laboratories (KCL), 8, Gaetbeol-ro 145beon-gil, Yeonsu-gu, Incheon, 21999, Korea

^5^Department of Life Science, University of Seoul, 163 Siripdaero, Dongdaemun-gu, Seoul 02504, Korea

^a^ Current address: Center for Nanosafety Metrology, Korea Research Institute of Standards and Science (KRISS), 267 Gajeong-Ro, Yuseong-Gu, Daejeon 34113, Republic of Korea

^b^ Current address: Ecology and Future Research Association, 21 Dusilro 45 beon-gil, Geumjeong-gu, Busan 46228, Korea

#: These authors contributed equally.

*Corresponding authors: Jinhee Choi (jinhchoi@uos.ac.kr) and Yun Doo Chung (ydchung@uos.ac.kr) of Univ. of Seoul and Tae-Soo Chon of Ecology and Future Research Association (tschon@pusan.ac.kr)

Total number of pages: 17

Total number of Tables: 6

Total number of Figures: 6

**Table S1**. Environment of animal exposure chamber (A) and toluene and formaldehyde concentration (B) in the inhalation chambers.

(A) Environment of animal exposure chamber in toluene and formaldehyde

| Treatment (ppm) | | Temperature (℃) | Humidity (%) | Pressure (mmH_2_O) | Air flow velocity (m/sec) |
| --- | --- | --- | --- | --- | --- |
| Toluene | 0 (Control) | 20.55 ± 0.003 | 46.34 ± 0.06 | -0.25 ± 0.005 | 174.29 ± 0.30 |
|  | 1 | 21.75 ± 0.002 | 47.33 ± 0.06 | -0.33 ± 0.007 | 174.29 ± 0.42 |
|  | 5 | 20.05 ± 0.001 | 45.84 ± 0.07 | -0.32 ± 0.011 | 174.27 ± 0.26 |
|  | 10 | 20.32 ± 0.004 | 47.04 ± 0.06 | -0.31 ± 0.011 | 174.33 ± 0.52 |
| Formaldehyde | 0 (Control) | 20.17 ± 0.17 | 57.87 ± 0.46 | -0.43 ± 0.006 | 170.58 ± 0.42 |
|  | 0.1 | 19.94 ± 0.20 | 58.29 ± 0.82 | -0.49 ± 0.012 | 170.60 ± 0.35 |
|  | 0.5 | 19.96 ± 0.08 | 58.51 ± 0.68 | -0.48 ± 0.003 | 170.31 ± 0.54 |
|  | 1.0 | 19.29 ± 0.07 | 61.70 ± 1.11 | -0.31 ± 0.011 | 170.06 ± 0.51 |

(B) Measured concentration of toluene and formaldehyde

| Treatment (ppm) | | Target Concentration  (mg/m^3^) | Measured concentration  (mg/m^3^) | Measured concentration  (μg/m^3^) |
| --- | --- | --- | --- | --- |
| Toluene | 0 (Control) | 0 | 0 | 0 |
|  | 1 | 1 ± 10 % | 1.11 ± 0.09 | 1110 ± 93.52 |
|  | 5 | 5 ± 10 % | 5.08 ± 0.28 | 5008 ± 283.05 |
|  | 10 | 10 ± 10 % | 10.25 ± 0.61 | 10250 ± 614.48 |
| Formaldehyde | 0 (Control) | 0 | 0 | 0 |
|  | 0.1 | 0.1 ± 10 % | 0.104 ± 0.004 | 104.38 ± 4.41 |
|  | 0.5 | 0.5 ± 10 % | 0.522 ± 0.022 | 522.45 ± 22.26 |
|  | 1.0 | 1.0 ± 10 % | 1.038 ± 0.041 | 1038.22 ± 40.90 |

**Table S2**. Differentially expressed genes (DEGs) in *Drosophila* affected by toluene (A) and formaldehyde (B) exposure (Based on 2-fold cutoff value). (See additional supplementary excel file)

**Table S3**. Gene ontology of DEGs. Biological process (A), Cellular Component and Molecular Function (B), GO terms) were sorted according to their enrichment p-value (< 0.05).

| (A) Biological process | | | | |
| --- | --- | --- | --- | --- |
| Toluene | | | | |
| Category | Term | Count | % | *P*-value |
| BP-UP | Innate immune response | 6 | 8.57 | 1.79E-05 |
|  | Response to heat | 5 | 7.14 | 9.96E-05 |
|  | Response to temperature stimulus | 5 | 7.14 | 1.31E-04 |
|  | Immune response | 6 | 8.57 | 3.24E-04 |
|  | Defense response | 6 | 8.57 | 3.61E-04 |
|  | Response to bacterium | 4 | 5.71 | 0.00298 |
|  | Proteolysis | 9 | 12.86 | 0.00371 |
|  | Response to abiotic stimulus | 5 | 7.14 | 0.00719 |
|  | Response to oxidative stress | 3 | 4.29 | 0.02065 |
|  | Defense response to bacterium | 3 | 4.29 | 0.02600 |
|  | Peptidoglycan catabolic process | 2 | 2.86 | 0.04651 |
|  | Peptidoglycan metabolic process | 2 | 2.86 | 0.04651 |
|  | Response to UV | 2 | 2.86 | 0.04651 |
| BP-DOWN | Antibacterial humoral response | 5 | 50.00 | 5.30E-10 |
|  | Defense response to bacterium | 5 | 50.00 | 2.60E-08 |
|  | Antimicrobial humoral response | 5 | 50.00 | 2.76E-08 |
|  | Response to bacterium | 5 | 50.00 | 4.75E-08 |
|  | Humoral immune response | 5 | 50.00 | 5.52E-08 |
|  | Immune response | 5 | 50.00 | 9.76E-07 |
|  | Defense response | 5 | 50.00 | 1.07E-06 |
|  | Innate immune response | 4 | 40.00 | 1.48E-05 |
|  | Defense response to Gram-negative bacterium | 2 | 20.00 | 0.01876 |
| Formaldehyde | | | | |
| BP-UP | Defense response | 28 | 14.21 | 6.12E-23 |
|  | Innate immune response | 22 | 11.17 | 1.53E-21 |
|  | Immune response | 26 | 13.20 | 1.20E-20 |
|  | Response to bacterium | 19 | 9.64 | 1.97E-18 |
|  | Defense response to bacterium | 18 | 9.14 | 3.52E-18 |
|  | Antibacterial humoral response | 11 | 5.58 | 4.06E-13 |
|  | Humoral immune response | 15 | 7.61 | 1.12E-12 |
|  | Antimicrobial humoral response | 14 | 7.11 | 2.09E-12 |
|  | Defense response to Gram-negative bacterium | 8 | 4.06 | 6.10E-08 |
|  | Aminoglycan metabolic process | 13 | 6.60 | 1.65E-07 |
|  | Polysaccharide metabolic process | 13 | 6.60 | 3.73E-07 |
|  | Toll signaling pathway | 7 | 3.55 | 1.51E-06 |
|  | Chitin metabolic process | 10 | 5.08 | 1.11E-05 |
|  | Aminoglycan catabolic process | 6 | 3.05 | 4.21E-05 |
|  | Polysaccharide catabolic process | 6 | 3.05 | 4.91E-05 |
|  | Proteolysis | 23 | 11.68 | 7.27E-05 |
|  | Defense response to Gram-positive bacterium | 5 | 2.54 | 2.00E-04 |
|  | Defense response to fungus | 5 | 2.54 | 2.76E-04 |
|  | Response to fungus | 5 | 2.54 | 3.71E-04 |
|  | Carbohydrate catabolic process | 6 | 3.05 | 0.00367 |
|  | Antifungal humoral response | 3 | 1.52 | 0.00649 |
|  | Regulation of Toll signaling pathway | 3 | 1.52 | 0.00937 |
|  | Peptidoglycan metabolic process | 3 | 1.52 | 0.01098 |
|  | Cell-matrix adhesion | 3 | 1.52 | 0.01098 |
|  | Peptidoglycan catabolic process | 3 | 1.52 | 0.01098 |
|  | Cell-substrate adhesion | 3 | 1.52 | 0.01098 |
|  | Glycosaminoglycan catabolic process | 3 | 1.52 | 0.01271 |
|  | Response to temperature stimulus | 5 | 2.54 | 0.01286 |
|  | Regulation of antibacterial peptide biosynthetic process | 3 | 1.52 | 0.02069 |
|  | Positive regulation of antibacterial peptide biosynthetic process | 3 | 1.52 | 0.02069 |
|  | Chitin catabolic process | 3 | 1.52 | 0.02069 |
|  | Regulation of antibacterial peptide production | 3 | 1.52 | 0.02069 |
|  | Response to cold | 2 | 1.02 | 0.02479 |
|  | Positive regulation of antimicrobial peptide biosynthetic process | 3 | 1.52 | 0.03557 |
|  | Regulation of antimicrobial peptide production | 3 | 1.52 | 0.03557 |
|  | Regulation of production of molecular mediator of immune response | 3 | 1.52 | 0.03557 |
|  | Regulation of antimicrobial peptide biosynthetic process | 3 | 1.52 | 0.03557 |
|  | Regulation of immune effector process | 3 | 1.52 | 0.03557 |
|  | Cell killing | 2 | 1.02 | 0.03696 |
|  | Killing of cells of another organism | 2 | 1.02 | 0.03696 |
|  | Response to oxidative stress | 4 | 2.03 | 0.04026 |
|  | Regulation of humoral immune response | 3 | 1.52 | 0.04718 |
|  | Regulation of response to biotic stimulus | 3 | 1.52 | 0.04718 |
|  | Glycosaminoglycan metabolic process | 3 | 1.52 | 0.04718 |
|  | Regulation of antimicrobial humoral response | 3 | 1.52 | 0.04718 |
|  | Regulation of multi-organism process | 3 | 1.52 | 0.04718 |
|  | Positive regulation of biosynthetic process of antibacterial peptides active against Gram-negative bacteria | 2 | 1.02 | 0.04898 |
|  | Regulation of biosynthetic process of antibacterial peptides active against Gram-negative bacteria | 2 | 1.02 | 0.04898 |
| BP-DOWN | Sensory perception of chemical stimulus | 4 | 18.18 | 0.00108 |
|  | Sensory perception | 4 | 18.18 | 0.00299 |
|  | Cognition | 4 | 18.18 | 0.00585 |
|  | Neurological system process | 4 | 18.18 | 0.01989 |
|  | Response to pheromone | 2 | 9.09 | 0.02135 |

| (B) Cellular Component & Molecular Function | | | | |
| --- | --- | --- | --- | --- |
| Toluene | | | | |
| Category | Term | Count | % | *P*-value |
| CC-UP | Extracellular space | 11 | 15.71 | 3.30E-12 |
|  | Extracellular region part | 11 | 15.71 | 1.58E-09 |
|  | Larval serum protein complex | 5 | 7.14 | 8.63E-09 |
|  | Extracellular region | 15 | 21.43 | 3.17E-08 |
|  | Lipid particle | 7 | 10.00 | 0.00143 |
| CC-DOWN | Extracellular space | 5 | 50.00 | 1.17E-06 |
|  | Extracellular region part | 5 | 50.00 | 1.36E-05 |
|  | Extracellular region | 6 | 60.00 | 0.00008 |
| MF-UP | Oxygen transporter activity | 6 | 8.57 | 1.82E-09 |
|  | Nutrient reservoir activity | 5 | 7.14 | 5.36E-09 |
|  | Peptidase activity | 10 | 14.29 | 0.00196 |
|  | Endopeptidase activity | 8 | 11.43 | 0.00395 |
|  | Serine-type endopeptidase activity | 6 | 8.57 | 0.00819 |
|  | Serine-type peptidase activity | 6 | 8.57 | 0.01207 |
|  | Serine hydrolase activity | 6 | 8.57 | 0.01239 |
|  | Peptidase activity, acting on L-amino acid peptides | 8 | 11.43 | 0.01850 |
|  | Hydrolase activity, acting on carbon-nitrogen (but not peptide) bonds, in linear amides | 3 | 4.29 | 0.03110 |
| Formaldehyde | | | | |
| CC-UP | Extracellular region | 40 | 20.30 | 2.38E-21 |
|  | Extracellular space | 17 | 8.63 | 8.71E-15 |
|  | Extracellular region part | 19 | 9.64 | 1.15E-12 |
|  | Larval serum protein complex | 4 | 2.03 | 5.32E-05 |
|  | Receptor complex | 4 | 2.03 | 0.01199 |
|  | Integral to plasma membrane | 8 | 4.06 | 0.02135 |
|  | Intrinsic to plasma membrane | 8 | 4.06 | 0.02298 |
| CC-DOWN | Extracellular region | 8 | 36.36 | 4.58E-06 |
| MF-UP | Endopeptidase activity | 21 | 10.66 | 1.92E-07 |
|  | Nutrient reservoir activity | 5 | 2.54 | 2.52E-07 |
|  | Serine-type endopeptidase activity | 16 | 8.12 | 6.57E-07 |
|  | Peptidase activity | 24 | 12.18 | 8.49E-07 |
|  | Polysaccharide binding | 11 | 5.58 | 1.50E-06 |
|  | Pattern binding | 11 | 5.58 | 1.50E-06 |
|  | Serine-type peptidase activity | 16 | 8.12 | 2.19E-06 |
|  | Serine hydrolase activity | 16 | 8.12 | 2.37E-06 |
|  | Peptidase activity, acting on L-amino acid peptides | 22 | 11.17 | 4.88E-06 |
|  | Oxygen transporter activity | 5 | 2.54 | 1.13E-05 |
|  | Carbohydrate binding | 11 | 5.58 | 6.83E-05 |
|  | Chitin binding | 8 | 4.06 | 1.51E-04 |
|  | Enzyme inhibitor activity | 8 | 4.06 | 2.44E-04 |
|  | Endopeptidase inhibitor activity | 7 | 3.55 | 3.15E-04 |
|  | Peptidase inhibitor activity | 7 | 3.55 | 3.84E-04 |
|  | Serine-type endopeptidase inhibitor activity | 6 | 3.05 | 9.05E-04 |
|  | N-acetylmuramoyl-L-alanine amidase activity | 3 | 1.52 | 8.17E-03 |
|  | Peptidoglycan binding | 3 | 1.52 | 9.58E-03 |
|  | Glycosaminoglycan binding | 3 | 1.52 | 0.02008 |
|  | Chitinase activity | 3 | 1.52 | 0.02008 |
|  | Structural constituent of peritrophic membrane | 3 | 1.52 | 0.02884 |
|  | Hydrolase activity, acting on carbon-nitrogen (but not peptide) bonds, in linear amides | 4 | 2.03 | 0.03367 |
| MF-DOWN | Pheromone binding | 3 | 13.64 | 0.00045 |
|  | Odorant binding | 4 | 18.18 | 0.00149 |
|  | Phosphatidylethanolamine binding | 2 | 9.09 | 0.01506 |

**Table S4**. List of genes and corresponding mutant stocks used for VOC toxicity test.

| **Biological pathway** | **Gene name** | **Genotype** | **Bloomington Stock #** | **Notes** |
| --- | --- | --- | --- | --- |
| Immune response | *ref(2)P* | *P{SUPor-P}ref(2)P^KG00926^* | 13287 |  |
|  | *imd* | *y^1^ w^67c23^; P{EPgy2}imd^EY08573^* | 17474 |  |
|  | *Traf6* | *w^1118^ P{EP}GIIIspla2^EP1516^ Traf6^EP1516^* | 17006 |  |
|  | *Tak1* | *w^*^ Tak^1179^* | 26275 |  |
| Stress response | *Dsor1* | *y^1^ w^*^ Dsor1^LH110^ P{FRT(whs)}101/FM7a* | 5545 | a |
|  | *rl* | *rl^1^* | 386 |  |
|  | *Mekk1* | *y^1^ w^67c23^; ry^506^ P{SUPor-P}Mekk1^KG02510^* | 13748 |  |
|  | *p38a* | *w^*^; P{ry[+t7.2]=neoFRT}82B p38a^1^* | 8822 |  |
|  | *p38b* | *y^1^ w^67c23^; P{y[+mDint2] w[BR.E.BR]=SUPor-P}p38b^KG01337^* | 14363 |  |
|  | *msn* | *w^*^; msn^102^ P{ry[+t7.2]=neoFRT}80B/TM6B* | 5945 | a |
|  | *bsk* | *bsk^1^ cn^1^ bw^1^ sp^1^/CyO* | 3088 | a |
|  | *puc* | *P{ry[+t7.2]=lArB}puc^A251.1F3^ ry^506^/TM3, Sb^1^* | 11173 | a |
|  | *Hsp23* | *w^1118^; P{w[+mGT]=GT1}Hsp23^BG01483^* | 12542 | b |
|  | *Hsp70Ab* | *y^1^ w^67c23^; P{w[+mC] y[+mDint2]=EPgy2}Hsp70Ab^EY01148^* | 15327 | b |
|  | *Hsp83* | *w^*^; Hsp83^e6D^/TM6B, Tb^1^* | 5696 | a |
|  | *Sod* | *Sod^n1^ red^1^/TM3, Sb^1^ Ser^1^* | 24492 |  |
|  | *p53* | *y^1^ w^1118^; p53^11-1B-1^* | 6816 |  |
| metabolism | *Cyp4e2* | *y^1^ w^67c23^; P{w[+mC] y[+mDint2]=EPgy2}Cyp4e2^EY09295^* | 17553 | b |
|  | *Cyp6d5* | *w^1118^; Mi{ET1}Cyp6d5^MB09381^ rdx^MB09381^* | 26475 | b |
|  | *GstD3* | *w^1118^; P{w[+mC]=XP}GstD3^d06796^/TM6B, Tb^1^* | 19250 | a |
|  | *GstE1* | *w^1118^; PBac{w[+mC]=RB}GstE1^e00657^* | 17876 | b |
|  | *GstS1* | *y^1^ w^67c23^; P{w[+mC]=lacW}GstS1^k08805^/CyO* | 10803 | a, b |

a: Heterozygous for the mutation; b: functional defects associated with this allele have not been proved yet.

**Table S5**. [Pearson correlation coefficients](http://www.baidu.com/link?url=i_e6fZ_q3tykV-vQPz9rF8CMhRlHLCmam0RFE-sMx6F2w74uxoEjHTtgSyd4PYZBreCDh7ikzimYxJIH8zN8BNdW85gVevnMkZ8NgDdN0M4urMkW9SNFo_OwS9L07Npi) between movement parameters.

|  | Speed  (mm/s) | Acceleration  (mm/s^2^) | Stop duration  (s) | Locomotory rate  (mm/s) | Meander  (rad/mm) | Slippping number  (n) | Fractal dimension |  |
| --- | --- | --- | --- | --- | --- | --- | --- | --- |
| Speed (mm/s) | 1.00 | - | - | - | - | - | - |  |
| Acceleration (mm/s^2^) | 0.87 ^**^ | 1.00 | - | - | - | - | - |  |
| Stop duration (s) | -0.95 ^**^ | -0.74 ^*^ | 1.00 | - | - | - | - |  |
| Locomotory rate (mm/s) | 0.75 ^*^ | 0.90 ^**^ | -0.65 | 1.00 | - | - | - |  |
| Meander (rad/mm) | -0.60 | -0.36 | 0.59 | -0.14 | 1.00 | - | - |  |
| Slippping number (n) | -0.67 ^*^ | -0.37 | 0.71 ^*^ | -0.18 | 0.96 ^**^ | 1.00 | - |  |
| Fractal dimension | 0.96 ^**^ | 0.86 ^**^ | -0.90 ^**^ | 0.66 | -0.64 | -0.66 | 1.00 |  |

(* indicates *p* < 0.05, while ** means *p* < 0.01)

**Table S6**. Gene and primer lists used for qRT-PCR analysis.

| Gene | Primer sequences |
| --- | --- |
| Gapdh | 5’ AGCTGGAGAAGGAAATGCTCAACG3’  5’ TGTCCTCCAGACCCTTGTTCTTCA3’ |
| *p38* | 5' ACGCACGCAGAAACTGTCTGATGA3' |
|  | 5' TCGTTTACCGCAATGTTCGATGGC3' |
| *P53* | 5' TCCAAGACGAACGCCAGCTCAATA3' |
|  | 5' TTTATAGCAATGCACCGACGCACC3' |


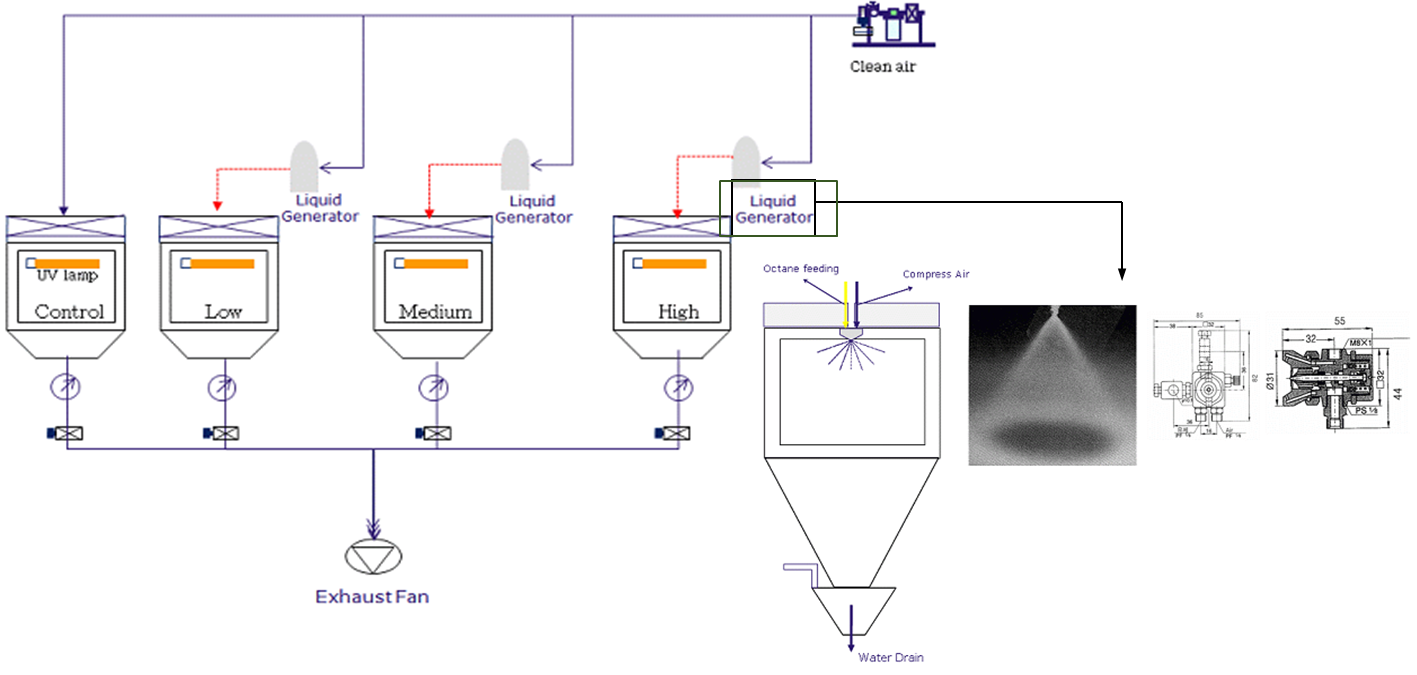


**Figure S1.** Diagram of inhalation toxicity study.


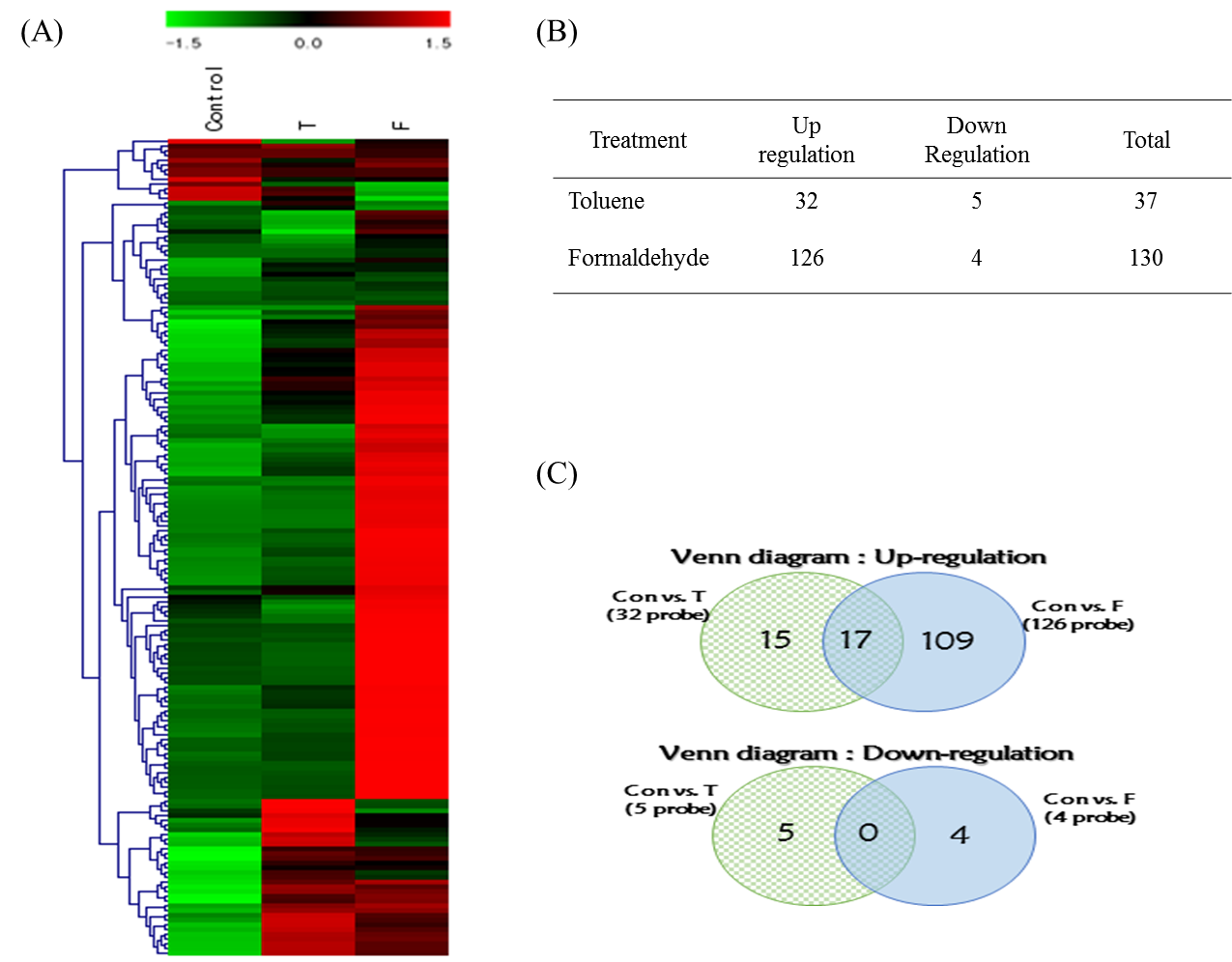


**Figure S2.** Microarray analysis. Hierarchical cluster (A), number of differentially expressed genes (B) and venn diagram of the number of differentially expressed genes (C). Microarray analysis was conducted on *Drosophila* exposed to toluene and formaldehyde for 24 h.

(A) (B)


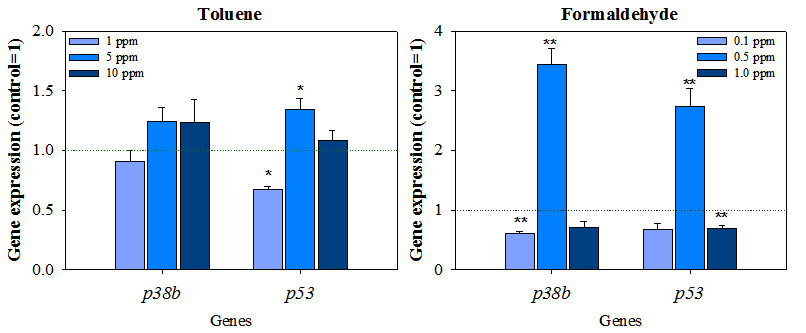


**Figure S3.** The expression of *p38b* and *p53* genes in wildtype exposed to toluene (A) and formaldehyde (B) using qRT-PCR. The results were expressed as the mean value compared to control (control=1, n=3; mean standard error of the mean). The statistical analysis was conducted using the two-tailed t-test, **p* < 0.05.

(A) (B)


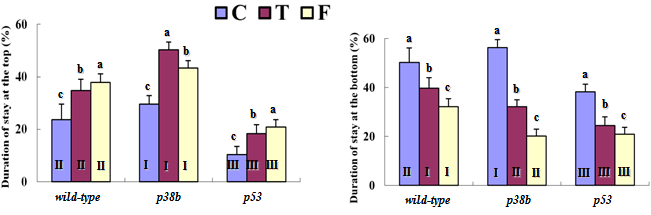


(C) (D)


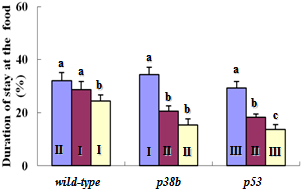


**Figure S4.** Mean and standard deviation of slipping number, and duration of stay in different areas in the observation arena when strains of *Drosophila* were treated with toluene and formaldehyde (n=15). Duration of stay at top (A), bottom (B), and food area (C). C: control; T: toluene; F: formaldehyde Different letters at the top of the bars indicate a significant difference between the chemicals within the same strain and different Roman numerals at the bottom of the bars shows a significant difference between the strains for the same chemical (*p* < 0.05) according to the multiple comparison tests.


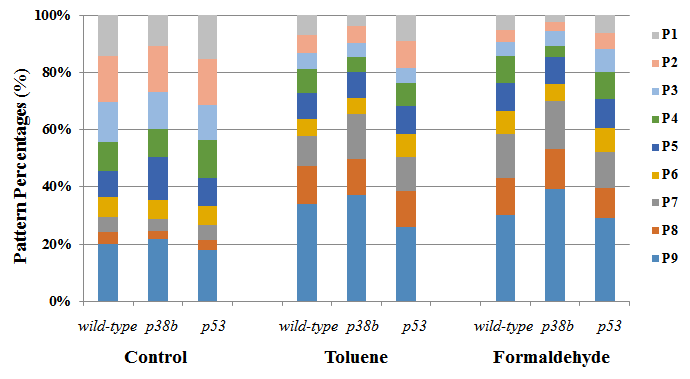


**Figure S5.** Percentage of movement patterns of *Drosophila* defined by SOM without and after the treatments (Patterns are listed in Fig. 5A)

(A)


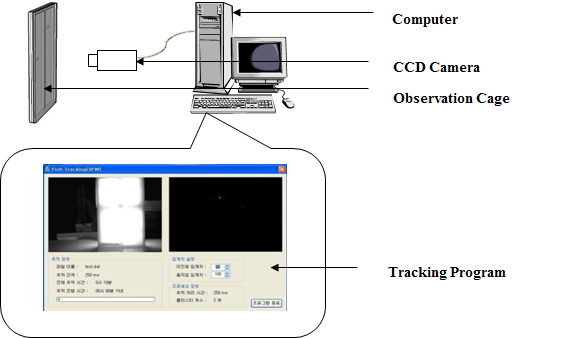


(B) (C)


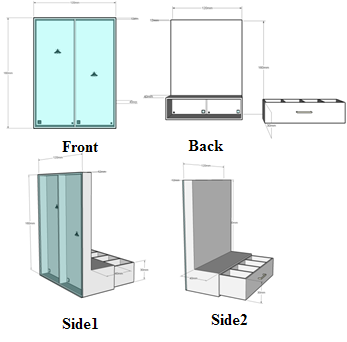

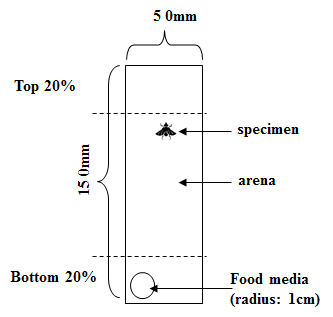


**Figure S6.** Behavior observation system for *Drosophila.* Tracking system (A), different views of observation cage (B), and subareas of observation cage (C).
